# Don’t speak too fast! Processing of fast rate speech in children with specific language impairment

**DOI:** 10.1371/journal.pone.0191808

**Published:** 2018-01-26

**Authors:** Hélène Guiraud, Nathalie Bedoin, Sonia Krifi-Papoz, Vania Herbillon, Aurélia Caillot-Bascoul, Sibylle Gonzalez-Monge, Véronique Boulenger

**Affiliations:** 1 Laboratoire Dynamique Du Langage, CNRS/Université de Lyon UMR5596, Lyon, France; 2 Service de Neurologie Pédiatrique, Hôpital Femme Mère Enfant, Bron, France; 3 Service Épilepsie, Sommeil et Explorations Fonctionnelles Neuropédiatriques, Hôpital Femme Mère Enfant, Bron, France; 4 Centre de Recherche en Neurosciences de Lyon, DYCOG, INSERM U1028 / CNRS UMR5292, Bron, France; 5 Service ORL chirurgie cervico-faciale, Centre Hospitalier Universitaire Gabriel Montpied, Clermont-Ferrand, France; 6 Centre de Référence Troubles des Apprentissages, Service de Rééducation pédiatrique, Hôpital Femme Mère Enfant, Bron, France; Max Planck Institute for Human Cognitive and Brain Sciences, GERMANY

## Abstract

**Background:**

Perception of speech rhythm requires the auditory system to track temporal envelope fluctuations, which carry syllabic and stress information. Reduced sensitivity to rhythmic acoustic cues has been evidenced in children with Specific Language Impairment (SLI), impeding syllabic parsing and speech decoding. Our study investigated whether these children experience specific difficulties processing fast rate speech as compared with typically developing (TD) children.

**Method:**

Sixteen French children with SLI (8–13 years old) with mainly expressive phonological disorders and with preserved comprehension and 16 age-matched TD children performed a judgment task on sentences produced 1) at normal rate, 2) at fast rate or 3) time-compressed. Sensitivity index (*d*′) to semantically incongruent sentence-final words was measured.

**Results:**

Overall children with SLI perform significantly worse than TD children. Importantly, as revealed by the significant Group × Speech Rate interaction, children with SLI find it more challenging than TD children to process both naturally or artificially accelerated speech. The two groups do not significantly differ in normal rate speech processing.

**Conclusion:**

In agreement with rhythm-processing deficits in atypical language development, our results suggest that children with SLI face difficulties adjusting to rapid speech rate. These findings are interpreted in light of temporal sampling and prosodic phrasing frameworks and of oscillatory mechanisms underlying speech perception.

## Introduction

Every listener has noticed how speech rate can vary considerably between speakers and contexts and how this can be particularly challenging, at least in the first minutes of a conversation. Speaking at a fast rate inevitably elicits qualitative changes in the speech signal at both temporal and spectral levels. With the increase in speech rate, length of acoustic cues, formant transitions and pauses are shortened [1]. Articulatory gestures are achieved more quickly and less accurately, resulting in reduction phenomena, enhanced coarticulation (i.e., increased gestural overlap) and assimilation, which may even lead to the suppression of whole segments [2]. Moreover, these changes operate nonlinearly, partly because of articulatory restrictions [3]: in English and French for instance, consonants and stressed syllables (in English) are less reduced than vowels and unstressed syllables [4,5].

Such spectro-temporal modifications typically occur in naturally accelerated speech, whereas the spectral and pitch content in time-compressed speech ‒ an artificial reduction of signal duration often used in experimental phonetics ‒ remain intact. Processing of naturally accelerated speech therefore puts high demands on the listener’s perceptual system to constantly normalize for a wide range of spectro-temporal parameters, in order to adapt to various speech rates and understand the message. The study by Janse [3] in adults highlighted the greater difficulty to process natural fast speech in comparison to time-compressed speech. Janse also compared two types of time compression: strict linear compression, where all syllables in the signal are compressed to the same degree to match the natural fast rate, and non-linear selective compression, which follows the exact temporal pattern (at the syllable level) of naturally produced fast speech. Results in a phoneme detection task showed a processing advantage for linearly time-compressed speech over both non-linearly time-compressed and natural fast speech. Linear time compression further led speech to be judged more pleasant to listen to. Hence, the increased segmental overlap combined with the changed temporal pattern which characterize natural fast speech make it more difficult to understand than artificially accelerated speech.

Studies focusing on the adaptation process involved in fast speech perception have revealed that young and older adults are able to adapt rather quickly to both types of accelerated speech (natural and artificial) [6–8]. For instance, Dupoux and Green [7] reported that listening to ten sentences compressed at 45% of their original duration was sufficient to improve performance in terms of the number of reported words in a sentence recall task. When sentences were made even faster (compressed at 38% of the original version), participants were still able to adapt, though after a slightly longer exposure time (15 sentences), therefore reflecting rapid perceptual adjustment even for highly accelerated speech. In a more recent study, Adank and Janse [6] compared adaptation to natural and artificial fast speech in young Dutch adults asked to perform a sentence verification task. Results revealed longer adaptation when listening to natural fast speech (exposure to 18 sentences was necessary) than to time-compressed speech (exposure to six to 12 sentences was sufficient). The same pattern of results, also consistent with the work of Janse [3], was observed in French-speaking typically developing children (aged 8–9 years; [9]), suggesting a qualitative difference between the processing of artificially accelerated speech (i.e., temporal change) and the processing of naturally-produced rapid speech (i.e., spectro-temporal changes). To account for adaptation processes to fast speech, it has been suggested that listeners must learn to associate new acoustic representations with stored phonemic categories. In other words, adaptation would require listeners to recalibrate (i.e., adjust) their phonemic boundaries according to those of the speaker, which could provide them with phonological representations that can be more easily processed by their perceptual system [9,10].

At the cortical level, one way this adjustment to varying speech rates can be achieved may entail oscillatory mechanisms. Studies using electro- and magnetoencephalography (E/MEG) have indeed shown that neuronal oscillations in the theta range (4–7 Hz), mainly in the right auditory cortex, synchronize to the slow modulations (3–5 Hz) dominating in the speech amplitude envelope, which mainly characterize syllabic rate (see [11] for a review). Such “entrainment” was observed for speech that was time-compressed until 50% of the original duration of the signal. When speech was no longer intelligible (at compression rates of 35% and 20%), cortical oscillations could however not align their phase to signal modulations [12]. These findings can be explained in the framework of the Asymmetric Sampling in Time (AST) model [13,14], which states that auditory cortices simultaneously sample the speech signal based on at least two temporal integration windows that correspond to the fundamental units of speech, namely phonemes and syllables. This temporal sampling is furthermore thought to occur asymmetrically. The left auditory cortex, on the basis of its spontaneous oscillatory activity lying around 40 Hz, may preferentially extract information from short integration windows (20–40 ms) and parse the signal into phonemic units (segmental level). In parallel, the right auditory cortex, naturally oscillating at approximately 4 Hz, would extract information from larger windows (150–250 ms) to process slower acoustic fluctuations in the amplitude envelope such as syllabic structure and prosodic cues (suprasegmental level). Segmental and suprasegmental information would then be integrated to access phonological representations.

The parsing of the continuous speech signal into segmental and suprasegmental features is of key importance for successful comprehension. Specifically, information about speech rhythm and linguistic stress carried by the temporal envelope is critical for intelligibility [11,15]. When speech is accelerated, the listener’s auditory system has to deal with faster temporal modulations in the continuous stream: it has to entrain to the new input rhythm to extract shortened units and to eventually enable efficient signal decoding. Accurate perception of rhythmic patterns conveyed by temporal changes in the amplitude envelope is also crucial for language acquisition, especially for the development of well-specified phonological representations [16]. In agreement with this, rhythm-processing deficits have been described in children with developmental dyslexia ‒ characterized by persisting major impairment in reading and spelling abilities [16] ‒ and in children with specific language impairment (SLI; note that SLI is now classified under the label “Language Disorder” as one form of neurodevelopmental communication disorder in the Fifth edition of the Diagnostic and Statistical Manual of Mental Disorders—DSM-5 [17], but see Bishop [18] for a discussion).

SLI, comprising 5 to 7% of the kindergarten and primary school population [19,20], is a specific, severe and long-lasting developmental language disorder, affecting the production and/or comprehension of spoken language, despite normal hearing development and non-verbal intelligence, and without any other cognitive or neurological deficits [21–23]. As children with SLI have been shown to present various linguistic symptoms, several classifications have been proposed to account for this heterogeneity. In France, clinicians mostly refer to the classification proposed by Rapin and Allen [24] who proposed three large sub-types of developmental disorders, including six profiles of language deficits that affect phonological, lexical, morpho-syntactic or pragmatic abilities. Mixed expressive-receptive disorders (including phonological-syntactic syndrome and verbal auditory agnosia) are distinguished from expressive disorders (verbal dyspraxia and speech programming deficit disorder) and from higher-order processing disorders (lexical deficit disorder and semantic-pragmatic disorder). Note that in this taxonomy, disorders are categorized according to the affected modality; however they may rather be seen as points on a continuum of language impairment than as discrete entities with clear boundaries between sub-types (see [25]).

Children with SLI have been shown to be impaired in non-verbal auditory discrimination tasks involving two important suprasegmental cues for speech rhythm and stress patterns, namely rise time of the amplitude envelope (i.e., rate of change in the envelope, corresponding to onsets of successive syllables, [26]) and signal duration [27]. Moreover, their rise time and duration processing abilities accounted for unique variance in several language and phonological tasks (see also [28]). These children however did not fail in non-verbal tasks that do not involve rhythm processing (e.g., intensity discrimination) as compared with chronological age-matched TD children. Note that reduced sensitivity to rise time variations has also been reported in dyslexic children [29], with or without associated oral language disorders [30]. A recent study in children with SLI [31] confirmed poor skills in discriminating rise time and duration, and these difficulties predicted performance in perceiving lexical stress patterns. In the so-called “temporal sampling framework” (proposed for dyslexia but encompassing SLI), Goswami [16] suggested that poor perception of the rhythmic structure in the speech envelope (i.e. slow modulations in speech) may compromise syllabic and word segmentation skills and consequently the formation of stable phonological representations. Cumming and collaborators [32] extended this notion to SLI with the “prosodic phrasing” hypothesis, emphasizing that perceptual difficulties for rhythmic cues such as rise time may also affect the extraction of prosodic patterns and lead to impaired comprehension as well as to morpho-syntactic impairments during language production.

Interestingly, rhythm-processing deficits in developmental language disorders have been shown to also occur at the motor level [29,33,34]. Children with SLI and dyslexia indeed exhibit difficulties tapping along in time to the slow tempo of a metronome (1.5 and 2 Hz, matching speech stress pattern). Again, children’s motor rhythmic performance can account for their phonological and literacy skills ([33] for SLI, [35] for dyslexia; see also [36] for evidence in pre-schoolers and [37] in adolescents). Recent works similarly demonstrated impaired entrainment to rhythm in nursery rhyme sentences or in music in dyslexic adults and children with SLI [34,38]. Timing deficits have also been described in children and adults with verbal dyspraxia both for the production of manual rhythms (e.g., clapped rhythm imitation and tapping) and of spoken rhythms (e.g., vocal rhythm reproduction) [39,40]. Beat synchronization implies fine auditory-motor synchrony in which precise timing information (i.e. sound’s onset) extracted in the auditory system is integrated with motor regions [41]. This also requires monitoring and appropriately adjusting the motor commands to match the auditory input. As previously mentioned, perception and production of rhythmic and temporal patterns are also crucial for language acquisition [16]. Auditory-motor coupling indeed plays a key role in the development of phonological skills as it requires the child to synchronize the timing of auditory perception with the motor networks involved in the representation and production of speech sounds [41]. Given these similarities, it has been suggested that a neural mechanism underlying the perception and expression of rhythm and timing, and involving auditory and motor brain regions [42,43], may be impaired in SLI and affect both language and motor development [33]. This is in agreement with the strong comorbidity observed between specific language and motor disorders in SLI [44], particularly when speech production is affected [45]. Most interestingly, structural and functional abnormalities have been described in SLI (verbal dyspraxia) in motor cortical and sub-cortical regions such as the cerebellum, the caudate nucleus and the supplementary motor area [46–48], regions that are not only involved in motor planning/sequencing and speech production but also in timing processing, both at the motor [43,49] and the linguistic levels [50–52].

Accumulating evidence therefore speaks in favour of impaired processing of acoustic cues to speech rhythm (i.e., stress and syllable prominence) and impaired rhythmic expression in children with SLI, which may impact prosodic and morpho-syntactic processes. The issue of how these children deal with natural speech processing when rhythmic temporal information of the ongoing signal is altered however remains to be tackled. As previously mentioned, amongst the multiple variations that speech can take in daily life, speech rate changes are ubiquitous both between and within speakers and between communication conditions. Given that speech rate is one of the temporal variables that characterizes speech rhythm, and given the deficits to process rhythmic information in children with SLI [26–28], examining the performance of these children to understand speech at varying syllabic rates may provide insights into their rhythmic abilities at the sentence level, in more ecological conditions. Increasing the rate of speech provides the listener with higher frequency modulations in the amplitude envelope, shorter segment durations and modified rise times due to the increased rate of change in the envelope (i.e. more frequent syllable “beats”). If children with SLI show poor perception of speech rhythmic temporal structure, accelerating the syllabic rate (and thus affecting this structure) may represent a challenge for these children and specifically affect their performance to understand speech. The present study set out to investigate the processing of fast rate speech in children with SLI showing difficulties mainly at the expressive level with preserved comprehension skills (phonological-syntactic syndrome and verbal dyspraxia) as compared with chronological age-matched children with typical development. The phonological-syntactic syndrome is the most prevalent form of SLI and is known to mainly affect verbal expression due a phonological programming disorder. The child is often hardly intelligible, shows altered phonology and tends to produce short and ungrammatical sentences [24,53,54]. Verbal dyspraxia affects the programming of articulatory movements despite intact neuromuscular system; impoverished phonological representations and phonological programming can also be part of the disorder [55]. Primary features of verbal dyspraxia include a restricted phonemic repertoire, inconsistency in articulation errors, difficulties in sequencing speech movements, prosodic abnormalities (affecting rate, rhythm, stress and intonation) and delayed expressive language [24,56]. In our experiment, children with predominant expressive disorders along the SLI continuum were included (i) because rhythmic processing deficits appear both at the acoustic and the motor levels (affecting auditory-motor coupling) and (ii) to ensure that they could perform the task and that their performance could not be merely attributed to poor general comprehension abilities.

We addressed this issue with a semantic judgment task on sentences either 1) naturally produced at a normal rate, 2) naturally produced at a fast rate or 3) artificially time-compressed (at the same rate as in 2). In line with the “prosodic phrasing” model [32], our hypothesis was that children with SLI may experience specific difficulty adjusting to fast temporal changes in the speech stream, which may reduce their performance in the sentence judgment task as compared with TD children. Including two types of rate acceleration furthermore allowed to determine whether children with SLI would benefit from the preservation of spectral content in time-compressed speech compared to natural fast speech (as was expected in TD children, see [3]), or whether they would be impaired as long as speech temporal structure is altered, leading to equally worse performance for artificial and natural acceleration. If children with SLI show a deficit to process speech rhythm [16,26–28,32], especially the modulations in the amplitude envelope which reflect syllabic rate, the latter pattern should be observed.

## Materials and methods

### Participants

Sixteen children with SLI mostly affecting the expressive modality (6 girls) aged 8–13 years old (mean = 10.9, SD 1.67) participated in the experiment. Each child was matched by age and sex with a typically developing (TD) child. All children with SLI were recruited through neuropsychologists and neurologists from a neuropediatric hospital unit where they had previously been diagnosed using standardized batteries of French verbal tests (e.g., ELO: *Evaluation du Langage Oral* [57]; N-EEL: *Nouvelles Epreuves pour l’Examen du Langage* [58]; BILO: *Bilans Informatisés de Langage Oral*) [59]) and non-verbal tests (WISC IV: Wechsler Intelligence Scale for Children [60]; WPPSI: Wechsler Preschool and Primary Scale of Intelligence) [61]). Verbal tests assessed phonological skills, production, morpho-syntax, vocabulary and oral comprehension. All children with SLI underwent their last neuropsychological evaluation (which confirmed the diagnosis) at the hospital within the six months preceding the experiment. Note that the referring neuropsychologists were not asked to provide any scores obtained by children in the different standardized tests. Their main involvement consisted of referring those children on their caseloads who met the criteria for inclusion in the study, by providing the parents of these children with an information flyer. The inclusion criteria (decided in collaboration with neuropsychologists and neurologists) encompassed age range from 8 to 13 years old, French native language, right-handedness, SLI with predominant expressive impairment, absence of hearing problem and non-verbal IQ > 70 (as recommended in the classification by Rapin and Allen [24]; see also [21,23,62,63] for a discussion on comparable language profiles between children with SLI with non-verbal IQ between 70 and 85 and children with SLI with IQ above 85). The exclusion criteria were bilingualism, mental retardation, the presence of attention-deficit/hyperactivity disorder (ADHD) and/or autism spectrum disorder. In our sample, thirteen children had been diagnosed with a phonological-syntactic syndrome affecting phonological programming (e.g., repetition and spontaneous language tested with the ELO [57]) and morpho-syntactic expression (e.g., sentence completion), whereas lexical processing and oral comprehension were preserved. Three other children had verbal dyspraxia with a disorder in orofacial motor programming affecting phonological production (one child had additional phonological deficits), combined with deficits in sensory-motor praxis (e.g., fingertip tapping, manual motor sequences and imitation of hand positions as assessed by sub-tests from the NEPSY, a developmental neuropsychological assessment [64]). To ensure the preservation of certain aspects of the diagnosis (expressive disorders with well-preserved comprehension and no mental retardation) at the time of the experiment, children’s performance was additionally assessed with a battery of neuropsychological and French language tests. Linguistic abilities were examined with sub-tests from the BALE (*Batterie Analytique du Langage Ecrit*; [65]). Non-verbal abilities were assessed with the Raven’s coloured Progressive Matrices [66] and the forward and backward digit span tests. Scores below 2 SD of the mean of the population were defined as pathological (see Table 1).

**Table 1:** Verbal and non-verbal abilities of children with SLI as assessed by standardized neuropsychological tests.

	Phonological-syntactic SLI	Verbal dypsraxia
Verbal tests		
Word repetition	**-5.25 (6.37)** (range -19.67 to 0.89)	-0.21 (0.64) (range -0.68 to 0.24)
Pseudo-word repetition	**-3.44 (3.13)** (range -8.33 to 0.43)	-1.42 (0.31) (range -1.64 to -1.20)
Non-word repetition	**-3.83 (3.39)** (range -10.67 to 0.60)	-1.17 (1.18) (range -2.00 to 1.10)
Phonemic fluency	-0.64 (0.95) (range -2.20 to 1.71)	-0.43 (2.17) (range -1.97 to 1.11)
Picture naming	-1.69 (2.97) (range -8.54 to 1.50)	0.4 (0.05) (range 0.36 to 0.43)
Expressive vocabulary (word definition)	-0.97 (0.95) (range -2.98 to 0.58)	-0.03 (0.74) (range -0.55 to 0.49)
Oral comprehension (sentence-to-picture matching)	-0.92 (1.28) (range -2.00 to 1.47)	-1.67 (0.06) (range -1.71 to 1.64)
Receptive vocabulary (word-to-picture matching)	-1.7 (1.43) (range -4.06 to 0.52)	-0.22 (0.08) (range -0.28 to -0.17)
Phonemic discrimination	**-2.5 (3.50)** (range -9.80 to 0.46)	-0.1 (0.50) (range -0.45 to 0.25)
Rhymes	-1.78 (1.79) (range -5.40 to 0.80)	-1.81 (0.84) (range -2.40 to -1.20)
Syllabic suppression	-1.29 (1.55) (range -4.50 to 0.85)	-0.96 (0.38) (range -1.24 to -0.69)
Phonemic suppression	-0.93 (1.18) (range -2.50 to 0.69)	-0.91 (1.05) (range -1.65 to 0.17)
Non-verbal tests		
Raven’s Progressive Matrices	89.61 (9.25) (range 71 to 103)	99 (11.31) (range 91 to 107)
Forward digit span	-0.82 (1.31) (range -2.60 to 0.80)	-0.15 (1.20) (range -1.00 to 0.70)
Backward digit span	-0.49 (0.88) (range -1.90 to 0.90)	-0.2 (0)

The typically developing children were recruited from elementary and secondary schools. All children were right-handed, French monolinguals and they were included in the study provided that they had no history of language and auditory deficit, nor of any other cognitive or neurological disorder (i.e. no deficit had ever been suspected by physicians, teachers or parents and no child had ever received language or cognitive treatment). Accordingly, neither IQ nor verbal abilities had been assessed with standardized batteries by (neuro)psychologists in these children (in France, IQ is tested in children by qualified clinical psychologists only when there is suspicion of a cognitive or intellectual deficit. IQ could not be assessed in our sample of TD children because of time constraints and most importantly because H.G. (who tested the children at school) is not allowed to administer this test as she is not a psychologist). Because of strong time constraints (the experiment was carried out during school time), non-verbal and verbal tests could not be administered to TD children; however, to guarantee typical language processing, we examined their (out loud) reading skills with the rapid (three minutes) French test “L’Alouette” [67] before the experiment. All children included in our experiment performed in the normal range (i.e. they were able to read the text within the allocated time without any mistakes): their reading age was comprised between 8.1 and 13.1, thus matching their chronological age range. This confirms general intact language abilities in these children. The protocol conformed to the Declaration of Helsinki and was approved by the local ethical committee (Comité de Protection des Personnes Lyon Sud-Est II; ID RCB: 2012-A00857-36). All children and their parents signed a consent form before the experiment.

Mean standard deviations for several sub-tests of the BALE [65], the Raven’s coloured Progressive Matrices [66] and the forward and backward digit span tests are reported for children with SLI with a phonological-syntactic syndrome and children with SLI with verbal dyspraxia. Standard errors as well as ranges for mean standard deviations are indicated in brackets. Tests for which children deviated from 2 SD from the mean of the population are highlighted in bold.

### Materials

Three hundred sentences (7–9 words) were created following the same syntactic structure: Determiner–Noun 1 –Verb–Determiner–Noun 2 –Preposition–Determiner–Noun 3. The semantic content of Noun 3 (disyllabic target word) was chosen to be contextually congruent with the beginning of the sentence in half of the sentences (n = 150) and incongruent in the other half (n = 150). Each target word appeared both in a congruent and an incongruent context (e.g., “Sa fille déteste la nourriture de la cantine” / *His daughter hates the food at the canteen* and “Le public applaudit le joueur pour sa cantine” / *The public applauds the player for his canteen*; see S1 File for examples). The semantic incongruity of the sentences was obvious as revealed by a pilot questionnaire proposed to twelve healthy adults. Sentence-final target words were controlled for lexical frequency, number of phonemes and number of phonological neighbours using the French lexical databases Lexique 3 [68] and Manulex (database on words in children books, [69]).

Sentences were recorded by a French native male speaker (44.1 kHz, mono, 16 bits) in a sound-attenuated booth using ROC*me*! Software [70]. Each sentence was recorded twice, at a normal and then at a fast rate. The procedure was the following: the sentence was first displayed on a computer screen in front of the speaker who was instructed to silently read it and to subsequently produce it aloud as a declarative statement at a normal rate. Once all sentences had been produced at a normal rate, they had to be produced at a faster rate using the same procedure. The speaker could produce each sentence several times so that the recorded version was as fluent as possible. The durations of the 2 × 300 sentences and the number of produced syllables for each sentence were then calculated with Praat software [71]. The average speech rate was 6.76 syllables/sec (SD 0.57) for natural normal rate sentences and 9.15 syllables/sec (SD 0.60) for natural fast sentences. Thus, the overall fast-to-normal ratio was 0.74 (i.e., speed-up factor of 1.35). Subsequently, the time-compressed sentences were computed by digitally shortening them with a PSOLA algorithm (Pitch Synchronous Overlap and Add [72]), as implemented in Praat. The compression rate was calculated for each sentence, and every individual time-compressed sentence was precisely matched by rate with its paired natural fast sentence. Compression was achieved by the re-synthesis of the normal rate stimulus, changing only the temporal structure without affecting the pitch. For the 900 sound files (300 × 3 rate variants), an 80 Hz high-pass filter was applied and the amplitude envelope was smoothed sentence-initially and finally. The intensity of the sound files was finally peak normalized.

The 900 sentences were divided into twelve experimental lists of 75 items each using a Latin square design so that each stimulus appeared in each rate condition across all participants but only once per list (to avoid repetition effects). No congruent/incongruent sentence pair was used within the same list. Sentence-final target words were matched for the above-mentioned psycholinguistic variables between lists. Each list was composed of three experimental blocks (25 items each, 13 semantically congruent/12 incongruent) corresponding to the three speech rate conditions. The blocks were always presented in the same order, namely normal rate sentences, then natural fast sentences and finally time-compressed sentences to avoid potential transfer of learning (see [10]). Each participant was presented with one of the twelve lists. Across the 12 lists, all target words were presented in the six different conditions (3 speech rates × 2 semantic incongruity). Within each experimental block, the order of the sentences was randomized across participants.

### Procedure

Children were comfortably seated in front of a laptop in a silent room and received oral instructions (the testing began once the experimenter ensured that children understood the instructions well). They were asked to attentively listen to the sentences and to perform a sentence semantic verification task. Each trial began with a white fixation cross presented at the centre of a black screen; after 1 s, the sentence was played diotically via headphones at a comfortable listening level, with the fixation cross remaining on the screen. The children then had to decide as quickly and accurately as possible whether the sentence made sense or not by pressing one of two pre-specified keys on the keyboard with their right index and middle fingers. Once they gave their response, the next trial was automatically played. If no response was given within 7 s, the trial was recorded as “no response” and the next trial was presented. Participants could listen to each stimulus only once. Before the testing phase, they were given five practice items (different from the experimental stimuli and produced by the same speaker at a normal or fast rate). The total duration of the experiment was 15 minutes. Stimulus presentation, response times and error measurements were performed using E-Prime 2 software (Psychology Software Tools, Inc., Pittsburgh, PA).

### Data analysis

Response times (RTs: time-interval between the onset of the sentence-final target word and the button press, in milliseconds) and accuracy (% of correct responses) were measured. Trials for which participants made no response or erroneous responses were considered as errors and were not included in RTs analysis. Trials with RTs below or above 2.5 standard deviations from the individual mean of the condition were further excluded from the analysis of RTs. We also computed *d*′ as an index of sensitivity to semantically incongruent sentence-final words and *ß* as an index of response bias. This calculation is based on the proportion of hits (i.e., correct responses for incongruent sentences, p[hits]) and false alarms (i.e., errors for congruent sentences, p[FAs]). *d*′ is defined as z(p[hits])-z(p[FAs]) and *ß* as z(p[hits])/z(p[FAs]) (see [73] for more details).

Before statistical analyses, the normality of the data was tested with the Shapiro-Wilk test. Both RTs and *d*′ followed a standard normal distribution (*W* = .98, *p* = .110 for RTs; *W* = .99, *p* = .407 for *d*′). A repeated-measures analysis of variance (ANOVA) was performed on RTs with Speech Rate (normal, fast, time-compressed) as the within-subject factor and Group (SLI, TD) as the between-subjects factor. As this analysis did not reveal any significant main effect nor interaction (*ps* > 0.1), the results on RTs are not presented here. A similar analysis was conducted on *d*′. In case of significant interaction, Tukey post-hoc comparison tests were performed. To estimate effect sizes, partial *η*^*2*^ were further calculated [74].

To assess the influence of age and non-verbal abilities (as assessed with the Raven’s Progressive Matrices in children with SLI) on children’s performance in the sentence judgment task, we carried out a mixed-effects linear regression with *d*′ as the dependent measure, Age and Raven’s scores as fixed factors and Participant as a random factor for each group (SLI and TD) and each speech rate condition (normal, natural fast and time-compressed).

Finally, as our group with SLI was composed of 13 children diagnosed with a phonological-syntactic syndrome and three children with verbal dyspraxia, we also performed the *d*′ analysis for the phonological-syntactic subgroup only (in order to check that the effects in the global analysis were not attributable to the subgroup with verbal dyspraxia, as not all classifications consider this disorder to be part of the SLI profile [75] contrary to the classification by Rapin and Allen [24] used in France). *d*′ did not follow a standard normal distribution in this subgroup (*W* = .98, *p* = .311); accordingly we used non-parametrical tests (Friedman chi-squared test, *Χ*^*2*^) including the factor Speech Rate, with post-hoc Wilcoxon test and Mann-Whitney test.

## Results

**Fig 1:**
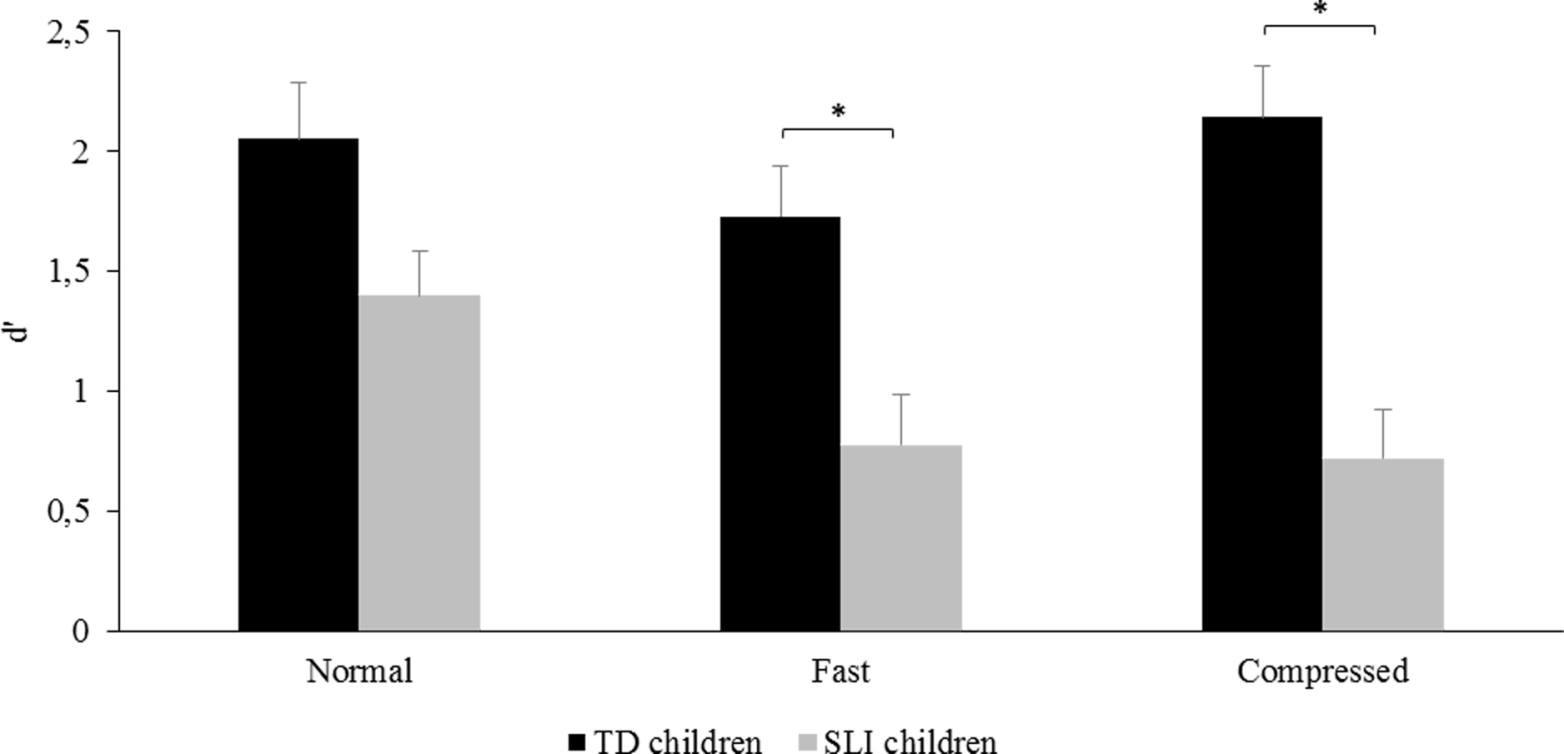
Children’s sensitivity to semantic incongruity during normal and fast rate speech processing. Mean *d′* values in children with SLI and in chronological age-matched typically-developing (TD) children are presented as a function of Speech Rate (natural normal, natural fast and time-compressed). (*) indicates a significant difference between conditions (*p* < .05). Error bars indicate standard errors.

The analysis on the *d*′ index revealed that children with SLI exhibited significantly reduced sensitivity to semantic incongruity in sentences (*d*′ = 0.96, standard error (SE) 0.09) as compared with TD children (*d*′ = 1.98, SE 0.05), *F*(1,30) = 15.47, *p* < .001, *η*_*p*_^*2*^ = .52. The main effect of Speech Rate was also significant, *F*(2,60) = 6.85, *p* < .003, *η*_*p*_^*2*^ = .23, with normal rate sentences leading to higher *d*′ values (*d*′ = 1.73, SE 0.22) than fast rate (*d*′ = 1.25, SE 0.24) and time-compressed sentences (*d*′ = 1.43, SE 0.28), although the differences did not turn out to be significant as assessed with post-hoc tests. Most importantly, Speech Rate significantly interacted with Group, *F*(2,60) = 4.50, *p* < .020, *η*_*p*_^*2*^ = .62, as illustrated in Fig 1. Tukey post-hoc tests revealed that children with SLI performed significantly worse than their TD age peers when listening to natural fast (*p* < .030) and time-compressed sentences (*p* < .001; Fig 1 and Table 1), whereas the difference between the two groups did not reach significance in the normal rate condition (*p* = .246).

The ANOVA on the *ß* index indicated a significant Group effect, *F*(1,30) = 26.66, *p* < .001, *η*_*p*_^*2*^ = .89, due to a more conservative decision bias in the TD group (*ß* = 1.41, SE 0.22) than in the SLI group (*ß* = 1.33, SE 0.21) (Table 2). There was no significant Speech Rate effect and no Speech Rate × Group interaction on the *ß* index.

**Table 2:** Mean d′ and β as a function of Group and Speech Rate.

	Mean *d′*			Mean *ß*		
Group	Normal	Fast	Time-compressed	Normal	Fast	Time-compressed
Children with SLI	1.31 (0.20)	0.71 (0.21)	0.63 (0.21)	1.52 (0.21)	1.07 (0.06)	1.36 (0.26)
TD children	2.12 (0.23)	1.77 (0.20)	2.22 (0.22)	1.43 (0.16)	1.06 (0.17)	1.59 (0.27)

Mean *d′* values are reported for children with SLI and age-matched TD children in the three speech rate conditions: natural normal, natural fast and time-compressed. Standard errors are indicated in brackets.

Results of the mixed-effects linear regression did not reveal any significant effect of Age or of non-verbal abilities (scores at the Raven’s Matrices), nor any interaction between the two, in any of the three speech rate conditions in children with SLI. The same analysis conducted in TD children with Age as a fixed factor also showed that this variable did not significantly affect *d*′ in the normal, natural fast and time-compressed conditions.

**Fig 2:**
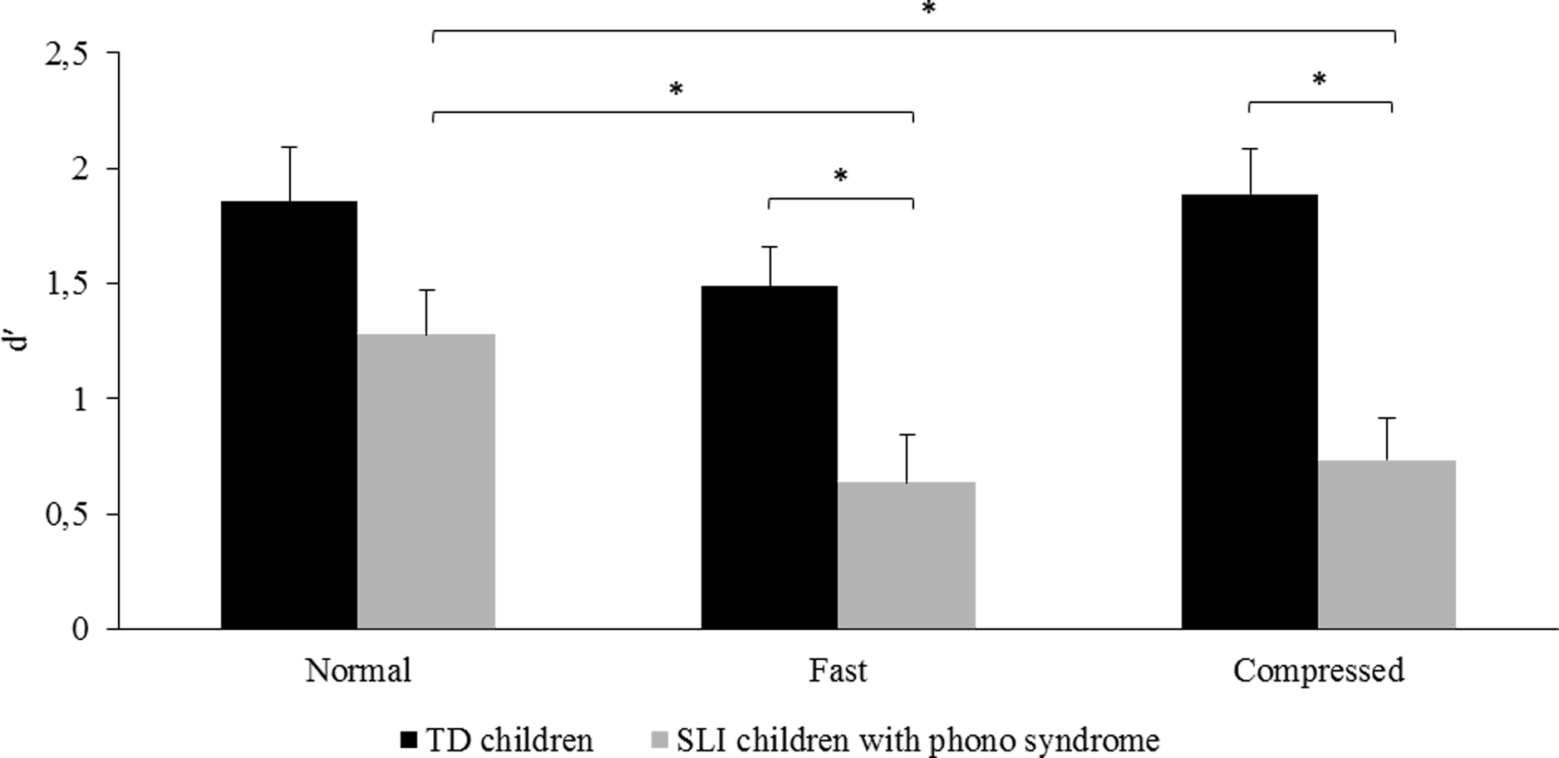
Sensitivity to semantic incongruity in children with SLI diagnosed with a phonological-syntactic syndrome as a function of Speech Rate. Mean *d′* values are presented for children with a phonological-syntactic syndrome (phono SLI) and chronological age-matched TD children for the three speech rate conditions (natural normal, natural fast and time-compressed). (*) indicates a significant difference between conditions (p < .05). Error bars indicate standard errors.

To make sure that the results presented above were not merely related to the inclusion of children with verbal dyspraxia, we performed a non-parametric analysis separately for the subgroup of children with a phonological-syntactic syndrome. The results are presented in Fig 2. The Friedman chi-squared analysis on the *d*′ index revealed a significant Speech Rate effect on children’s performance, *Χ*^*2*^(2) = 7.54, *p* < .023. Sensitivity to sentence semantic incongruity was higher in the normal rate condition (*d*′ = 1.28, SE 0.20) than in the natural fast (*d*′ = 0.64, SE 0.21, *V* = 76.00, *p* < .040) and the time-compressed conditions (*d*′ = 0.73, SE 0.18, *V* = 82.00, *p* < .009) as revealed by a Wilcoxon post-hoc test; the latter two conditions did not significantly differ from each other. The same analysis in the group of 13 paired TD children showed that the effect of Speech Rate only approached significance, *Χ*^*2*^(2) = 5.69, *p* = 0.058 (Fig 2), mainly reflecting a trend towards higher sensitivity in the normal rate condition (*d*′ = 1.85, SE 0.24) than in the natural fast rate condition (*d*′ = 1.49, SE .17; *V* = 71.00, *p* = .080 as shown by the Wilcoxon test). To compare the performance of the two groups (TD children and children with a phonological-syntactic syndrome) in each condition, *U* Mann-Whitney tests were carried out. Analyses highlighted that children with a phonological-syntactic disorder were less sensitive to semantic incongruity in sentences than their TD peers in the fast (*W* = 30.50, *p* < .007) and time-compressed conditions (*W =* 20.00, *p* < .002). In the normal rate condition, no significant difference was found between the two groups (*W* = 51.50, *p* = .100), thus confirming the results obtained when all children with SLI were included in the analysis (Fig 1).

Friedman chi-squared tests on the *ß* index in each group of children did not reveal any significant difference between speech rate conditions. However, a significant Group effect emerged when analysing the three conditions separately with *U* Mann-Whitney tests (*W* normal = 6.00, *p* < .001; *W* fast = 20.00, *p* < .001; *W* time-compressed = 22.00, *p* < .002), reflecting a more conservative bias decision in the TD group (*ß* = 1.40, SE 0.20) than in the group with a phonological-syntactic syndrome (*ß* = 1.31, SE 0.18). Note that this was also observed in the analysis including all children with SLI.

## Discussion

The present study investigated the processing of fast rate speech in children who suffer from SLI mainly at the expressive level and in paired children with typical development (TD). Speech processing was assessed through a semantic judgment task performed on aurally presented sentences at either natural normal, natural fast or time-compressed rate. The results revealed that children with SLI were less sensitive to semantic incongruity than TD children, and that this sensitivity varied with speech rate. Crucially, the interaction between Group and Speech Rate highlighted that the deficit of the SLI group regarding semantically incongruent word detection was significant only in the case of rapid speech signals. It is therefore more challenging to detect semantic incongruity in sentences for children with SLI than for their TD age-peers when speech is accelerated, either naturally or artificially. No difference emerged between the two groups when sentences were produced at a normal rate.

Children with two profiles of language impairment mainly affecting the expressive modality, namely phonological-syntactic syndrome and verbal dyspraxia [24], participated in the experiment. To ascertain that the observed results were not related to the inclusion of children with verbal dyspraxia, we also analyzed the data in the sub-group of children with a phonological-syntactic syndrome only. The results replicated those found when all children with SLI were included: children with a phonological-syntactic syndrome were indeed less sensitive to semantic incongruity in sentences than TD children when speech was naturally or artificially accelerated. This suggests that the general findings cannot only be attributed to the inclusion of children with verbal dyspraxia in our experimental sample, and that they are clearly consistent with the performance of children diagnosed with a phonological-syntactic syndrome. Note that in our experiment, children were aged between 8 and 13 years old; given this rather wide age range, performance in the sentence judgment task may thus be assumed to vary with age. The regression analysis including age as a fixed factor did not reveal any significant influence of this variable on performance in the TD and SLI groups. However, it may be important for future studies including a larger number of participants to systematically compare age ranges (i.e., 8–10 vs. 11–13 years old) to investigate the developmental trajectory of fast rate speech processing in these children. Besides, in children with SLI, no significant influence of non-verbal abilities (as assessed with the Raven’s Progressive Matrices) was found on sensitivity to semantic incongruity in this analysis. Although this needs to be more thoroughly assessed with more participants for a well-powered analysis, this suggests that performance of children with SLI was not affected by their perceptual intellectual abilities.

In the present study, a cut-off value of 70 for non-verbal IQ was determined as an exclusionary criterion for children with SLI based on the classification proposed by Rapin and Allen [24] used by clinicians (see also for instance [76]). This may be considered rather low compared to most other studies using international classifications where only children with SLI with a non-verbal IQ higher than 85 (i.e., 1 standard deviation) are included in order to avoid confounds created by intellectual deficits (note however that 13 out of our 16 children with SLI had scores above 85 at the Raven’s Progressive Matrices at the time of the experiment). The issues of whether a large discrepancy between verbal and non-verbal abilities is needed and whether to extend the exclusion criterion to an IQ of 70 or above have been recently debated in the literature [21,77,78]. As pointed out by Parisse and Maillart [23], the strict integrity of non-verbal skills in SLI seems very demanding and not well justified. First, the same pattern of language deficit can characterize children with low (≤85) or high (>86) non-verbal IQs [20]. Second, studies have shown that children with SLI with higher non-verbal IQ do not benefit more from language interventions than children with SLI with lower skills [79–81]. Third, non-verbal IQ can drop or fluctuate considerably over time in SLI with a decline up to 20 points (see [82] for a review), possibly due to persistent language impairment which makes it difficult to obtain non-verbal scores in the normal range. Altogether, this casts doubt on the meaning of non-verbal IQ of 85 in SLI [77] and makes us believe that in the present study, children with SLI did not perform poorer for fast rate sentences because of their non-verbal skills.

To our knowledge, our findings provide the first piece of evidence for the impact of speech rate on sentence understanding in children, consistent with previous effects shown in adults [6]. Speeding up the rate of speech, by requiring the listener to cope with temporal changes (as well as with spectral ones in the case of natural acceleration) [3], is thus more demanding and can impact sentence-level integration processes. The processing of fast speech becomes even more problematic for children with SLI, even when their developmental language disorders predominantly affect verbal expression with rather preserved comprehension skills, as diagnosed by neuropsychologists with standardized tests. This result suggests that neuropsychological measurements of receptive linguistic skills in children should include more ecological tests using fast speech material to assess the actual difficulties experienced by young patients in their daily life.

It has been previously reported that natural fast speech is more difficult to understand than time-compressed speech, mostly due to increased gestural overlap that occurs only when speech is naturally accelerated [6,9]. Although pointing toward this effect, the present pattern of results does not provide clear support for this difference in children. Nevertheless, qualitative inspection of Fig 1 suggests that TD children tended to perform slightly better when listening to time-compressed speech as compared with natural fast speech, which was not the case for the group of children with SLI. Care has to be taken in interpreting non-significant results, but one tentative explanation for this observation is that, unlike children with typical development, children with SLI do not take advantage of the preservation of spectral cues in the case of artificially time-compressed speech and are impaired from the moment syllabic rate is accelerated. Another potential explanation is that, in the context of this experiment, only TD children benefitted from listening to natural fast sentences to subsequently process time-compressed speech. Such transfer of learning has been reported in adults [6], however it occurred in the reverse order, from the artificial to the natural condition. This was interpreted according to the Reverse Hierarchy Theory [83] which suggests that skills learned in an easier condition (i.e., time-compressed speech) can be subsequently applied to a more complex one (i.e., natural fast speech) but not the reverse. In this view, listening to time-compressed speech would imply learning at high processing levels, then enabling to focus attention on lower-level, more specific cues and to subsequently adapt to natural fast speech. In the present study, natural fast speech was presented before time-compressed speech to avoid such a bias. Our data suggest slightly better processing of time-compressed than of natural fast speech in TD children and show poorer-than-normal performance to decode both naturally and artificially accelerated speech in children with SLI. Nevertheless, it is not altogether impossible that some kind of learning occurred in TD children. An experiment varying the order of presentation of the two types of fast speech in two sub-groups of participants would be necessary to more precisely assess transfer of learning in TD and children with SLI.

One could argue that the low performance of the SLI group to achieve the task in our experiment may result from overall limited speed of information processing. Several studies have indeed shown longer reaction times to sentence-embedded words in SLI, thought to reflect limitations in real-time computing of cognitive operations. Stark and Montgomery [84] for instance reported that children with SLI were slower than age-matched controls in detecting target words in spoken sentences, independently of speech rate. In the same word-monitoring paradigm, Montgomery [85] demonstrated lengthened reaction times in these children as compared with TD children for immediate processing of fast rate sentences, whereas the reverse pattern was observed for slower rate stimuli, suggesting that language-impaired children need more time to accurately complete the required cognitive operations. In our study, children with SLI exhibited significant lower sensitivity to semantic incongruity with increased speech rate as compared with TD children, however no significant difference emerged between the two groups regarding response times. This finding is at odds with the results by Montgomery [85] and speaks against processing speed limitation as the main cause of the deficit to process natural fast and time-compressed speech in our SLI children. Furthermore, in our study, the lack of any significant interaction between Group and Speech Rate on the response criterion *ß* suggests that this is not due to decisional strategies. Note also that the task was different from that used in the aforementioned studies [84,85] where children had to remember a target word and provide a timed response immediately upon recognition of this word within a sentence. In our experiment, children had to make a semantic judgment about the entire sentence. Although one could suggest that reduced *d*′ in children with SLI stems from their poor understanding of fast linguistic information, we remind the reader that our sentence material was carefully selected in accordance with the children’s age (see Method) to ensure comprehension. The children with SLI were furthermore mainly impaired at the expressive level rather than at the receptive level, and their non-verbal intelligence was also satisfactory, as diagnosed by neuropsychologists and assessed with the Raven’s Progressive Matrices. The decrease in performance observed in the SLI group ‒ especially for natural fast and time-compressed speech ‒ reported here can therefore not be interpreted as merely reflecting semantic impairment nor altered non-verbal reasoning skills. To fully rule out the semantic explanation, future studies on fast rate speech processing in children with SLI should yet make use of other tasks tackling phonological (e.g., sentence-embedded phoneme or syllable identification) and morpho-syntactic processing (e.g., grammaticality judgment). In addition, examining whether performance of children with SLI for fast rate speech in these tasks correlates with (and can be predicted by) auditory and motor rhythmic abilities (e.g., rise time and duration perception, finger tapping, beat perception) would allow characterizing the rhythmic nature of the observed deficit. This would also discard the alternative interpretation that less efficient perceptual processing, as is seen for speech-in-noise perception [86–89], underlies poorer-than-normal fast rate speech processing in SLI. Note though that the comparable performance of children with SLI for artificial and natural acceleration does not seem to favor this interpretation which would predict lower performance for natural fast (i.e. spectro-temporal changes) than for time-compressed sentences (i.e. temporal changes).

Our pattern of results is in agreement with the Rapid Auditory Processing (RAP) theory [90,91], suggesting that children with SLI primarily suffer from deficient temporal processing of sequential brief acoustic cues, such as brief complex tones separated by short temporal intervals or phonemes that acoustically differ only by rapid transient formants (e.g., /b/ vs. /d/). Improved speech processing was for instance observed in children with SLI when transitions were lengthened [91]. Accordingly, the authors concluded that RAP deficits in SLI may lead to ill-formed phonological representations and thus to atypical language development. In this view, poorer performance of our SLI group for natural fast and time-compressed speech could therefore stem from difficulties dealing with shortened phonemic units in the sentences. However, along with the deficit for very brief cues themselves, children’s poor performance could also reflect a deficit to process long ongoing sequences of short segments such as in the fast sentences used in our experiment. Rapid succession of brief acoustic cues particularly affects children with SLI, who have been shown to present with temporal processing deficits [33,92]. As a matter of fact, increasing speech rate does not only reduce the duration of acoustic cues but it additionally affects the dynamics of connected speech, in particular the low-frequency amplitude modulations which characterize signal temporal structure and syllabic rhythm.

Rhythm is a hallmark for speech communication [93,94] and accurate perceptual sensitivity to acoustic rhythmic information in speech is fundamental for language development [36,95]. Processing of rhythm entails, besides the identification of short cues, the detection of temporal regularities in the unfolding signal in order to predict upcoming relevant events such as onsets of syllables, words or phrases. Such anticipation of events’ occurrence may imply a temporal shift of attention. According to the Dynamic Attending Theory [52,96], attention is not distributed uniformly over time but it is periodic, with high levels of energy oriented towards salient external events. Self-sustained internal oscillators would synchronize (or entrain) to external sensory rhythms, therefore generating temporal expectancies on the occurrence of future events whose temporal integration would then be optimized. Crucially, attentional rhythms are assumed to adapt their phase and period to the rate changes in external rhythms so as to maintain synchronicity [52,96]. As far as speech is concerned, temporal characteristics are mainly conveyed by slow amplitude fluctuations which are approximately the duration of syllables and appear at rather regular and thus predictable intervals. The listener’s internal oscillators can therefore track this quasi-periodicity and align their phase to that of the amplitude envelope, allowing to anticipate the onset time of forthcoming syllables. This is supposed to facilitate speech segmentation and comprehension ([11] for a review) and to also occur when the rate of speech is accelerated.

As mentioned in the Introduction, deficits in the perception and expression of rhythm have been reported in atypical language development [16,27,31–33]. Children with SLI may therefore be impaired in detecting temporal regularities in external sensory stimuli, which could explain why language-impaired children in the present study performed worse than their TD peers when listening to accelerated speech. Poor entrainment to rapid speech rhythm may actually impede efficient extraction of syllable onsets to parse the speech stream into key units for decoding. Uttering speech at a fast rate alters signal temporal structure (as well as spectral content for natural acceleration), which is reflected in the amplitude envelope by faster (i.e., higher frequency) and smaller (i.e., less sharp) modulations than in clear, well-articulated speech, as well as by shorter acoustic cues such as rise time [97,98]. When exposed to accelerated speech, the listener’s auditory system has therefore to entrain to more frequent syllable “beats” [12] to ensure accurate speech segmentation and decoding, a process thought to be fundamental for language acquisition as it promotes prosodic processing [99]. Accordingly, our results of poorer performance for both naturally and artificially accelerated speech in children with SLI may be interpreted as reflecting their lower ability to adjust to the faster syllabic rhythm as compared with TD children, therefore affecting their performance in a sentence judgment task. The present study does not currently allow disentangling between the rapid auditory processing hypothesis [90,91] and this rhythmic tracking interpretation, which however may not exclude one another. One attempt to do so would be to use the paradigm developed by Ghitza and Greenberg [100] with normal rate sentences, accelerated sentences (i.e., shorter speech segments and faster syllabic rhythm) and accelerated sentences with periodic insertion of silences so as to restore the original sentence rhythm (i.e., shorter segments but normal rhythm). Following our current results, children with SLI should perform poorer for fast rate sentences; however, if their deficit stems from difficulties in tracking speech temporal structure (more frequent syllable beats), they should improve when silences are inserted periodically and restore sentence’s temporal dynamics. On the contrary, if their deficit is mainly due to impaired processing of shortened speech units, inserting silences should have no beneficial effect in these children. Additionally including slowed speech (i.e., longer segments and slower rhythm) and slowed speech with silences (i.e., normal length segments and slower syllabic rhythm) would also be interesting to investigate the processing speed issue as well as to potentially provide clinical recommendations for SLI.

Regarding the rhythmic interpretation, our findings may be explained in the context of multi-time resolution models of speech processing [13,14] suggesting that phase-locking between theta (4–7 Hz) oscillations in auditory cortex and slow fluctuations in the amplitude envelope is critical for intelligibility [11]. This alignment between brain rhythms and speech rhythms, already present in infants [101,102], is assumed to guarantee efficient detection of syllable prominence and therefore reliable speech understanding [103,104]. EEG/MEG studies have revealed abnormal patterns of low-frequency oscillatory activity in auditory regions during processing of rhythmic noise or speech stimuli in adults with developmental dyslexia [105,106] and in children with poor reading skills, especially for time-compressed speech [107]. To the best of our knowledge, no such investigation has been conducted in children with SLI so far; however, impaired processing of speech rhythm in these children may be expected to result, at least partly, from atypical functional neural entrainment to slow modulations in speech, hence hindering syllabic parsing and prosodic processing [32]. As suggested by our results, this may be even more evident for fast rate speech perception.

As rhythm-processing deficits arise at the motor level as well, oscillatory abnormalities could furthermore not be circumscribed to auditory cortical regions but could spread to articulatory regions. Perception of acoustic rhythm, in music and also in speech, implies tight reciprocal coupling between temporal auditory and frontal motor planning regions [42,43]. In agreement with the Dynamic Attending Theory [96], such interactions would enable predicting upcoming beats and therefore enhance rhythmic input processing [41,108]. At the neuroanatomical level, auditory-motor coupling may be mediated by the dorsal stream, which connects posterior temporal to premotor regions *via* the inferior parietal cortex [109,110]. This pathway is thought to play a key role in language development and to underlie speech sensorimotor integration, particularly under compromised acoustic conditions [111,112]. At the functional level, communication between auditory and motor areas could operate through oscillatory synchrony [111]. At rest, alignment between endogenous oscillations in temporal and premotor cortices has indeed been described in theta and gamma ranges, two frequency bands relevant for syllabic and phonemic sampling respectively [113]. Interestingly, Lehongre and coworkers [114] further reported reduced low-gamma (25–35 Hz) entrainment to amplitude-modulated white noise in auditory but also in left articulatory motor and somatosensory regions in dyslexic adults. Accordingly, one may hypothesize that impaired oscillatory dynamics, most likely in the theta syllabic range, within the dorsal sensorimotor network also exists in children with SLI, affecting their abilities to process speech rhythm. As the dorsal stream is specifically involved in the perception of degraded speech (e.g., time-compressed or noisy speech, [115,116]), potential abnormal neural communication between auditory and premotor cortices in children with SLI may be even more detrimental to the perception of fast rate speech. Future investigations are nevertheless needed to examine brain auditory-motor tracking of speech at various rates together with rhythmic abilities in children with SLI.

To the best of our knowledge, our study is the first to compare the perception of fast rate speech, accelerated naturally or artificially, in French speaking children with SLI. Results reveal that these children perform worse than their TD age peers to decode both natural fast and time-compressed speech (i.e. as soon as syllabic temporal information is speeded up), suggesting poor entrainment to amplitude fluctuations that characterize speech rhythm. These findings, consistent with previous works on rhythm-processing deficits in developmental language disorders [31,32], provide arguments that maintaining a normal rate when talking to children with language disorder, in classrooms but also during neuropsychological assessment and remediation, is essential to help speech processing and communication. Besides, processing of fast speech in children with SLI could take advantage of the recent line of research on the impact of musical interventions on language processing. Growing evidence indeed points towards the benefit offered by rhythmic stimulation and music on phonological and morpho-syntactic abilities in typically developing children but also in children with dyslexia or SLI [92,117,118]. If poor decoding of fast rate speech in children with SLI results, at least partly, from an inability to accurately adjust to accelerated speech rhythm, entrainment to musical rhythms may be expected to improve subsequent language performance in these children (see [119]).

## Supporting information

S1 File Stimulus audio files. Example sentences are provided in the three speech rate conditions (natural normal rate, natural fast rate and time-compressed; sentence rate is indicated in syllables per second) and the two semantic conditions (congruent and incongruent).
(ZIP)

S1 Table Participants’ data. Means for the different behavioral measures (response times (RT), scores, hits, false alarms (FA), *d′*, c, β) are reported for children with SLI and typically-developing (TD) children in each speech rate condition. Scores for the Raven’s Progressive Matrices are also reported for children with SLI (note that data is missing for one child as it was not possible for him/her to undergo the additional tests due to fatigue; however, this child had a perceptual reasoning index (as assessed by clinicians) in the normal range).
(DOCX)
