# Supplementary material for: Don’t speak too fast! Processing of fast rate speech in children with specific language impairment
Source: PLoS One. 2018 Jan 26;13(1):e0191808. doi: 10.1371/journal.pone.0191808 (PMC5786310; doi:10.1371/journal.pone.0191808)
Supplement: S1 Table — Means for the different behavioral measures (response times (RT), scores, hits, false alarms (FA), d′, c, β) are reported for children with SLI and typically-developing (TD) children in each speech rate condition. Scores for the Raven’s Progressive Matrices are also reported for children with SLI (note that data is missing for one child as it was not possible for him/her to undergo the additional tests due to fatigue; however, this child had a perceptual reasoning index (as assessed by clinicians) in the normal range). (DOCX) [file pone.0191808.s002.docx]

|  | Semantically-congruent sentences | Semantically-incongruent sentences |
| --- | --- | --- |
| Example 1 | Les détectives trouvent un indice pour leur enquête (*The detectives find an evidence for their inquiry*) | Un gardien surveille l’entrée de l’enquête (*A guard watches the entrance of the inquiry*) |
| Natural normal rate | File1.wav  6.59 syll/s | File2.wav  6.04 syll/s |
| Natural fast rate | File3.wav  9.13 syll/s | File4.wav  8.34 syll/s |
| Time-compressed | File5.wav  9.13 syll/s | File6.wav  8.34 syll/s |
| Example 2 | Sa fille déteste la nourriture de la cantine (*His daughter hates the food at the canteen*) | Le public applaudit le joueur pour sa cantine (*The public applauds the player for his canteen*) |
| Natural normal rate | File 7.wav  6.02 syll/s | File 8.wav  6.36 syll/s |
| Natural fast rate | File9.wav  8.60 syll/s | File10.wav  9.60 syll/s |
| Time-compressed | File11.wav  8.60 syll/s | File12.wav  9.60 syll/s |
